# Supplementary material for: Modeling glioblastoma heterogeneity as a dynamic network of cell states
Source: Mol Syst Biol. 2021 Sep 16;17(9):e10105. doi: 10.15252/msb.202010105 (PMC8444284; doi:10.15252/msb.202010105)
Supplement: Supplementary file 6 — Source Data for Figure 5 [file MSB-17-e10105-s004.zip › Figure5A_sourcedata/GSEA_3071/hallmarks_stateI.GseaPreranked.1623742531165/HALLMARK_INTERFERON_GAMMA_RESPONSE.html]

Details for gene set HALLMARK\_INTERFERON\_GAMMA\_RESPONSE[GSEA]

|  || Dataset | state13071 |
| Phenotype | NoPhenotypeAvailable |
| Upregulated in class | na\_pos |
| GeneSet | HALLMARK\_INTERFERON\_GAMMA\_RESPONSE |
| Enrichment Score (ES) | 0.50967574 |
| Normalized Enrichment Score (NES) | 2.6795177 |
| Nominal p-value | 0.0 |
| FDR q-value | 0.0 |
| FWER p-Value | 0.0 |
Table: GSEA Results Summary

  

Fig 1: Enrichment plot: HALLMARK\_INTERFERON\_GAMMA\_RESPONSE      
 Profile of the Running ES Score & Positions of GeneSet Members on the Rank Ordered List

  

| PROBE | GENE SYMBOL | GENE\_TITLE | RANK IN GENE LIST | RANK METRIC SCORE | RUNNING ES | CORE ENRICHMENT || 1 | SSPN |  |  | 23 | 1.574 | 0.0436 | Yes |
| 2 | CFH |  |  | 29 | 1.523 | 0.0908 | Yes |
| 3 | TNFAIP6 |  |  | 36 | 1.470 | 0.1361 | Yes |
| 4 | NMI |  |  | 131 | 0.962 | 0.1396 | Yes |
| 5 | APOL6 |  |  | 178 | 0.859 | 0.1537 | Yes |
| 6 | SAMD9L |  |  | 180 | 0.857 | 0.1808 | Yes |
| 7 | B2M |  |  | 241 | 0.744 | 0.1872 | Yes |
| 8 | IFITM3 |  |  | 268 | 0.714 | 0.2025 | Yes |
| 9 | IRF1 |  |  | 276 | 0.705 | 0.2230 | Yes |
| 10 | LGALS3BP |  |  | 286 | 0.696 | 0.2426 | Yes |
| 11 | TAPBP |  |  | 287 | 0.692 | 0.2647 | Yes |
| 12 | SOCS1 |  |  | 292 | 0.686 | 0.2855 | Yes |
| 13 | ISG15 |  |  | 328 | 0.647 | 0.2960 | Yes |
| 14 | EPSTI1 |  |  | 394 | 0.592 | 0.2961 | Yes |
| 15 | TNFSF10 |  |  | 395 | 0.590 | 0.3149 | Yes |
| 16 | IFI44L |  |  | 422 | 0.574 | 0.3257 | Yes |
| 17 | PSMB9 |  |  | 437 | 0.560 | 0.3396 | Yes |
| 18 | NAMPT |  |  | 456 | 0.544 | 0.3518 | Yes |
| 19 | DDX60 |  |  | 481 | 0.527 | 0.3616 | Yes |
| 20 | NCOA3 |  |  | 514 | 0.499 | 0.3683 | Yes |
| 21 | BPGM |  |  | 546 | 0.481 | 0.3747 | Yes |
| 22 | IFI35 |  |  | 574 | 0.467 | 0.3818 | Yes |
| 23 | CASP4 |  |  | 585 | 0.460 | 0.3936 | Yes |
| 24 | MTHFD2 |  |  | 588 | 0.459 | 0.4077 | Yes |
| 25 | VCAM1 |  |  | 603 | 0.450 | 0.4180 | Yes |
| 26 | IL15 |  |  | 635 | 0.439 | 0.4230 | Yes |
| 27 | PSMB8 |  |  | 669 | 0.423 | 0.4270 | Yes |
| 28 | BST2 |  |  | 677 | 0.421 | 0.4384 | Yes |
| 29 | ADAR |  |  | 709 | 0.408 | 0.4425 | Yes |
| 30 | IFI44 |  |  | 753 | 0.396 | 0.4427 | Yes |
| 31 | IFIT3 |  |  | 767 | 0.393 | 0.4515 | Yes |
| 32 | IFITM2 |  |  | 836 | 0.370 | 0.4436 | Yes |
| 33 | KLRK1 |  |  | 842 | 0.367 | 0.4539 | Yes |
| 34 | PSMA3 |  |  | 866 | 0.361 | 0.4587 | Yes |
| 35 | RBCK1 |  |  | 879 | 0.356 | 0.4666 | Yes |
| 36 | JAK2 |  |  | 886 | 0.353 | 0.4762 | Yes |
| 37 | IFIH1 |  |  | 962 | 0.336 | 0.4652 | Yes |
| 38 | STAT2 |  |  | 976 | 0.333 | 0.4720 | Yes |
| 39 | CASP1 |  |  | 981 | 0.331 | 0.4815 | Yes |
| 40 | ZNFX1 |  |  | 1013 | 0.325 | 0.4829 | Yes |
| 41 | PARP14 |  |  | 1026 | 0.323 | 0.4897 | Yes |
| 42 | RNF213 |  |  | 1027 | 0.322 | 0.5000 | Yes |
| 43 | LY6E |  |  | 1030 | 0.321 | 0.5097 | Yes |
| 44 | MX1 |  |  | 1130 | 0.302 | 0.4906 | No |
| 45 | STAT1 |  |  | 1136 | 0.301 | 0.4988 | No |
| 46 | SOCS3 |  |  | 1172 | 0.294 | 0.4981 | No |
| 47 | ARL4A |  |  | 1195 | 0.289 | 0.5009 | No |
| 48 | PML |  |  | 1211 | 0.286 | 0.5057 | No |
| 49 | PARP12 |  |  | 1244 | 0.279 | 0.5054 | No |
| 50 | MX2 |  |  | 1282 | 0.271 | 0.5033 | No |
| 51 | UBE2L6 |  |  | 1370 | 0.256 | 0.4863 | No |
| 52 | PSMB10 |  |  | 1375 | 0.256 | 0.4933 | No |
| 53 | CASP3 |  |  | 1639 | -0.273 | 0.4258 | No |
| 54 | CD274 |  |  | 1966 | -0.314 | 0.3414 | No |
| 55 | CDKN1A |  |  | 2083 | -0.332 | 0.3183 | No |
| 56 | PDE4B |  |  | 2132 | -0.339 | 0.3153 | No |
| 57 | PNP |  |  | 2223 | -0.358 | 0.3006 | No |
| 58 | ST3GAL5 |  |  | 2526 | -0.421 | 0.2266 | No |
| 59 | ARID5B |  |  | 2826 | -0.511 | 0.1563 | No |
| 60 | MT2A |  |  | 2910 | -0.547 | 0.1497 | No |
| 61 | SRI |  |  | 3177 | -0.757 | 0.0968 | No |
Table: GSEA details [plain text format]

  

Fig 2: HALLMARK\_INTERFERON\_GAMMA\_RESPONSE: Random ES distribution      
 Gene set null distribution of ES for **HALLMARK\_INTERFERON\_GAMMA\_RESPONSE**
